# Accurate and efficient representation of intramolecular energy in *ab initio* generation of crystal structures. Part III: partitioning into torsional groups

**DOI:** 10.1107/S2052520624010072

**Published:** 2025-01-22

**Authors:** Isaac J. Sugden, David H. Bowskill, Benjamin I. Tan, Yizu Zhang, Claire S. Adjiman, Constantinos C. Pantelides

**Affiliations:** a Department of Chemical Engineering, Sargent Centre for Process Systems Engineering, Institute for Molecular Science and Engineering, Imperial College London, LondonSW7 2AZ, United Kingdom; University of Geneva, Switzerland

**Keywords:** crystal structure prediction, computational chemistry, flexible molecules, energy partitioning

## Abstract

We present an approach to reduce this computational cost substantially, based on the partitioning of the molecule into geometrically separated torsional groups, with the dependence of the intramolecular energy and atomic point charges and dependent degrees of freedom on molecular conformation being computed as a linear combination of the contributions of these groups. This can lead to large savings in computational cost without a significant impact on accuracy, as demonstrated in the cases of *N*-acetyl-*para*-amino­phenol (paracetamol) and methyl 4-hy­droxy­benzoate (methyl paraben). The approach is also applied successfully to two larger molecules, benzyl [4-(4-methyl-5-[(4-methyl­phenyl)­sulfonyl]-1,3-thia­zol-2-yl)phenyl]­carbamate (molecule XX from the fifth CSP blind test) and (2*S*)-2-[4-(3-fluoro­benzyl­oxy)benzyl­amino]­propionamide (safinamide), for which we conduct the first reported CSP study. In both cases, the use of torsional groups results in over 99% reduction in computational cost, which enables the generation of an initial CSP landscape with high-quality structures found within the standard cutoff of 20 kJ mol^−1^ for progression to refinement.

## Abstract 2

An algorithm for dramatically reducing the number of QM calculations required to derive a flexibility model of molecules, whilst retaining the accuracy necessary for Crystal Structure Prediction, is presented. Torsional group partitioning can make previously inaccessibly large molecules tractable, and molecule 26 from the blind tests, and safinamide, are demonstrated as use cases

## 1. Introduction

Crystal structure prediction (CSP) methods seek to provide a short, yet complete, list of experimentally realizable crystal structures for a given molecule or set of molecules, solely based on knowledge of the relevant molecular connectivity diagram(s). The *ab initio* determination of the crystalline structures that a molecule can form could be very impactful for several industrial sectors, enabling the faster development of manufacturing processes and de-risking the production, distribution and storage of crystalline products. For the pharmaceutical industry, for instance, a key motivation is avoiding the catastrophic withdrawal of a drug resulting from the appearance of a previously unknown, less soluble, polymorph, as was the case for ritonavir (Chemburkar *et al.*, 2000 ▸), DPC 961 (Rietveld & Céolin, 2015 ▸) and rotigotine (Rietveld & Céolin, 2015 ▸; European Medicines Agency, 2005 ▸). There are likely many more examples of solid form issues affecting drug product development that are not in the public domain, as pharmaceutical companies are motivated to exhaustively explore the solid form landscape by these cases (Lee *et al.*, 2011 ▸).

The field of crystal structure prediction has witnessed a great deal of progress in recent years, as shown by the increasing size and flexibility of targets in the blind tests organized by the Cambridge Crystallographic Data Centre (CCDC) (Day *et al.*, 2009 ▸; Bardwell *et al.*, 2011 ▸; Reilly *et al.*, 2016 ▸). In CSP methods, it is generally assumed that viable crystals are low-energy local minima with respect to the cell variables [lengths and angles, molecule position(s) and orientation(s)], and any internal (conformational) degrees of freedom. In most cases, given the small contribution of entropic effects to the total free energy, coupled with the high cost of evaluating entropic contributions, lattice energy is assumed to suffice for the ranking of crystal structures (Bowskill *et al.*, 2021 ▸; Francia *et al.*, 2020 ▸; Abraham & Shirts, 2020 ▸).

Most successful CSP methodologies use a multistage approach (Day *et al.*, 2009 ▸; Bowskill *et al.*, 2021 ▸), in which a large set of candidate structures is initially generated using a relatively simple energy model, followed by successive refinements of the most promising (lowest energy) candidates carried out using increasingly accurate models. This stage-wise framework has been widely adopted because, for the molecules that have typically been studied, covering the full breadth of possible crystal structures necessitates 10^5^–10^6^ minimizations in the initial global search step, which would render the use of the most accurate and expensive energy models prohibitively expensive. It is, therefore, essential that the initial global search uses a lattice energy model that is computationally efficient enough to perform millions of minimizations, whilst being sufficiently accurate to ensure that the low-energy forms are not excluded from the next refinement step. Several approaches have been proposed to derive a lattice energy model, or potential, all of which include some degree of customization to the molecule(s) of interest (Bowskill *et al.*, 2021 ▸).

The lattice energy model used at this stage could include a general force field, such as the Dreiding (Mayo *et al.*, 1990 ▸), Compass (Sun *et al.*, 1998 ▸) or Charm (MacKerell *et al.*, 2000 ▸) forcefields, as was common in the first blind test of crystal structure prediction (Lommerse *et al.*, 2000 ▸). This approach has not generally been successful in blind tests, as the energy model is not tailored to the molecule of interest, and the subtle differences in stability between crystals structures are not described accurately enough for relevant crystal structures to be progressed to the more computationally intense ranking stages. The improvement of individual force field parameters by tailoring with Quantum Mechanical (QM) calculations has a long precedent (Schmidt *et al.*, 2007 ▸), and extending this methodology to using QM calculations on crystal structures, and extending this methodology to using QM calculations on all the low energy crystal structures, often termed the Ψ_crys_ method (Price, 2018 ▸), has been shown to be a successful approach under blind test conditions. The program *GRACE* (Neumann *et al.*, 2008 ▸) for instance, has been very successful (Reilly *et al.*, 2016 ▸), and makes use of tailor-made force fields in which the traditional force field terms (angle bends, intramolecular van der Waals terms *etc*.) are parameterized by QM calculations. A similar approach is used in the more recent methodology adopted in *XtalPi* (Zhang *et al.*, 2018 ▸). Such approaches require access to very significant high performance computing resources [*e.g.* in the sixth blind test, groups making use of periodic DFT methods routinely, required millions CPU hours for a given molecule (Reilly *et al.*, 2016 ▸)] and, due to this high cost, can often only be deployed with relatively simple levels of theory/basis set, leading to potential inaccuracies or incomplete global searches (Nyman *et al.*, 2019 ▸). Another class of methods, often termed the Ψ_mol_ method (Price, 2018 ▸), consists in tailoring some of the force field parameters to QM calculations on the isolated molecule, while using transferable parameters for some terms in the force field. Such an approach, initially adopted by van Eijck *et al.* (2001 ▸) and in *DMACRYS* (Price *et al.*, 2010 ▸), has been further developed in the *CrystalPredictor* suite of global search codes (Karamertzanis & Pantelides, 2005 ▸, 2007 ▸; Habgood *et al.*, 2015 ▸; Sugden *et al.*, 2016 ▸, 2019 ▸) and in the rigid-molecule global search code GLEE (Case *et al.*, 2016 ▸; Yang & Day, 2022 ▸). These approaches have been successfully applied in some challenging global searches (Beran *et al.*, 2022 ▸; Kazantsev, Karamertzanis, Adjiman, Pantelides *et al.*, 2011 ▸). *CrystalPredictor II* (Habgood *et al.*, 2015 ▸; Sugden *et al.*, 2016 ▸, 2019 ▸) has been applied successfully to a wide range of systems (Wade *et al.*, 2022 ▸; Tchoń *et al.*, 2021 ▸; Schmidt *et al.*, 2021 ▸; Racher *et al.*, 2023 ▸; Pawlak *et al.*, 2021 ▸; Braun *et al.*, 2019 ▸; Shunnar *et al.*, 2020 ▸; Braun *et al.*, 2021 ▸), including flexible molecules and co-crystals (Sugden *et al.*, 2022 ▸). In the latter case, it has proven to be especially efficient as isolated-molecule QM calculations can be reused in modelling a co-crystal, thereby avoiding numerous calculations. Nevertheless, the applicability of *CrystalPredictor II* to molecules with more than six to seven flexible torsions is limited as the computational cost increases exponentially with the number of such torsions. To address this, this manuscript focuses on developments for the program *CrystalPredictor*, improving its efficiency further without compromising its accuracy. In the *CrystalPredictor II* energy model there are no traditional forcefield terms beyond repulsion/dispersion, and minor internal degrees of freedom such as angle bends are updated as a function of the major degrees of freedom such as torsions, as will be described.

In a variation of an approach first suggested by van Eijck *et al.* (2001 ▸), *CrystalPredictor II* uses isolated-molecule QM calculations to create customized models for the intramolecular energy, selected (dependent) conformational variables and the electrostatic potential as functions of a set of independent degrees of freedom that usually comprises the most flexible torsion angles. The concept of independent/dependent variables is a mathematical construct, rather than a chemical description, and reduces the computational cost without compromising accuracy. These tailored models are referred to as Local Approximate Models (LAMs) (Kazantsev *et al.*, 2010 ▸) and are initially constructed by performing QM calculations at fixed values of the independent degrees of freedom (LAM reference points) placed on a uniform grid (Habgood *et al.*, 2015 ▸). Conformationally dependent properties (energy, electrostatic potential, dependent degrees of freedom) are then approximated via Taylor expansions around the nearest LAM reference point. It has been shown that, within their area of validity, LAMs provide near-QM accuracy for the conformationally dependent properties (Sugden *et al.*, 2016 ▸), at a much reduced computational cost relative to a full QM calculation. Recent advances have allowed the bulk of the computational effort to be focused in areas of crystallographic relevance through an adaptive LAM algorithm (Part I: Sugden *et al.*, 2016 ▸), and have led to improvements in efficiency and accuracy through the introduction of a smoothed LAM potential (Part II: Sugden *et al.*, 2019 ▸).

However, a difficulty with this approach is that the number of grid points required rises exponentially with the number of independent degrees of freedom. Even a relatively coarse uniform grid can become very expensive to evaluate for molecules with five to six flexible torsions and completely impractical for more flexible molecules. For example, Ritonavir has 22 independent degrees of freedom (Chemburkar *et al.*, 2000 ▸); an extremely coarse grid with just three points in each dimension would involve approximately 31 billion grid points, each requiring an isolated-molecule QM calculation. Moreover, in practice, a much finer grid might be required to achieve a sufficiently good level of accuracy in energy evaluations.

In many pharmaceutically relevant molecules, however, the flexible torsion angles are often separated geometrically in *para* or *meta* positions of rigid benzyl groups. For example, a search of the Crystal Structure Database (CSD; Groom *et al.*, 2016 ▸) (November 2021 version) reveals that 170549, or 46.08%, of 370142 di-substituted benzene moieties were in the *para* arrangement, with 23467 (6.34%) in the *meta* and 176126 (47.58%) in the *ortho* arrangements. Our hypothesis is that, for compounds with *para* or *meta* arrangements, the cross interactions between torsion angles with such geometrical separation are likely to be negligible. As we will see later in this paper, this hypothesis makes the investigation of larger and more flexible molecules tractable. It should be pointed out that this approximation would not hold for molecular connectivities without geometric separation, such as aliphatic chains.

Partitioning flexible degrees of freedom into (approximately) non-interacting groups was a concept previously used in *CrystalPredictor I* which, instead of LAMs, used restricted Hermite interpolants to approximate the intramolecular energy and electrostatic potential. While this was successful for several systems (Francia *et al.*, 2021 ▸; Iuzzolino, 2018 ▸; Kazantsev, Karamertzanis, Adjiman, Pantelides *et al.*, 2011 ▸), the applicability of this approach had been limited by the requirement that the molecule’s dependent degrees of freedom , *i.e.* its bond lengths, bond angles and non-flexible torsions, be fixed to their *in vacuo* values. This often leads to conformations with unnecessarily high intramolecular energy values. Furthermore, because the use of restricted interpolants can lead to a sharp decrease in the quality of the approximation as the number of degrees of freedom increases, there is a practical limit of three torsions per group to maintain accuracy.

The above shortcomings are addressed by the more recent LAM-based approaches employed by *CrystalPredictor II*. However, the number of flexible degrees of freedom that can be handled is still limited by computational cost. This is the issue that the current paper aims to address via the introduction of torsional angle groups. In Section 2, we present the algorithmic framework, briefly re-examining the concept of local approximate models (LAMs) and their effect on conformationally dependent properties, and outlining the changes that need to be introduced to the lattice energy model to account for torsional group partitioning. In Section 3, we investigate the impact of the use of partitioning on the accuracy of the LAMs, using methyl­paraben and molecule XX of the fifth CSP blind test (Bardwell *et al.*, 2011 ▸) as test cases. In Section 4, the approach is tested in the context of CSP by conducting global searches for four molecules with the modified *CrystalPredictor II* algorithm and analysing the results. The findings are summarized in Section 5.

## 2. LAMs with partitioning

The global search stage of CSP as implemented in *CrystalPredictor II* is preceded by (i) the identification of the main flexible torsions (independent degrees of freedom) in the isolated molecule(s) of interest; (ii) the generation of a database of LAM reference points, each involving an isolated-molecule QM minimization for the corresponding fixed values of the independent degrees of freedom, followed by the generation of a local approximate model for the corresponding optimal values of the remaining molecular geometry variables (the dependent degrees of freedom), the electrostatic potential of the molecule(s) and the intramolecular energy. During the global search itself, a large number of candidate crystal structures are generated using a quasi-random sequence, and each one of them is used as a starting point for a local minimization of the lattice energy using a gradient-based algorithm. The pre-generated LAMs are used to calculate the intramolecular energy contributions to the lattice energy, the atomic positions within the unit cell, which are needed to compute all intermolecular energy contributions, and the atomic charges used in the electrostatic energy contributions.

The focus of the current paper is in achieving a significant reduction in the cost of LAM generation for large, flexible molecules. We therefore present this step in more detail next.

### 2.1. Standard formulation of LAMs

In order to construct a LAM, the molecule’s conformational degrees of freedom are partitioned into a vector of *l* independent degrees of freedom, θ, and a vector of *M*  dependent degrees of freedom, 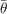, where θ contains the flexible torsions that have the largest impact on the intramolecular energy *U*^intra^. The intramolecular energy for given values of θ is then determined via the solution of an *in vacuo* energy minimization,
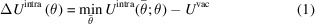
where the semicolon indicates that the independent degrees of freedom θ are kept constant during the optimization, and *U*^vac^ is the minimum *in vacuo* energy of the molecule, obtained by solving the following problem:
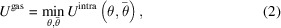
where the values of the optimization variables at the solution are denoted by θ^vac^, 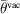.

In addition to computing the minimum intramolecular energy for the given values of θ, the QM minimization (2) also yields the corresponding values of the dependent degrees of freedom and the electrostatic potential field.

The above QM calculations are performed at a set of *N*_ref_ distinct reference values of θ, denoted by 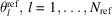. The combination of a second-order Taylor expansion of the intramolecular energy and the optimality conditions of equation (2) leads to the following LAMs (Kazantsev, Karamertzanis, Adjiman & Pantelides, 2011 ▸) that are valid in the vicinity of reference point 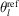:

(*a*) intramolecular energy difference, Δ*U*^intra^,

(*b*) dependent conformational degrees of freedom, 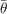 and

(*c*) point charges, *q*.

Details of each LAM are provided in the supporting information.

More recently, a smoothed LAM (Sugden *et al.*, 2019 ▸) based on a weighted average of the LAM expressions across several reference points has been introduced to obtain continuously differentiable approximants for the intramolecular energy, dependent degrees of freedom and point charges. The developments presented here are equally applicable to LAMs based on a single reference point or on a weighted average.

Regardless of the approach chosen, all reference points used in *CrystalPredictor II* are pre-computed using a uniform (Habgood *et al.*, 2015 ▸) or adaptive (Sugden *et al.*, 2016 ▸) grid and stored in a database, which can be accessed during local lattice energy minimizations. Where a conformation is indicated to have a lower energy than the current *U*^vac^, the θ^vac^, 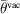 and *U*^vac^ are updated. As can be seen in equations (1) and (2), at each reference point, a QM minimization is carried out and the Hessian matrix at the solution is computed. As a result, the generation of the LAM database is often the most expensive component of the global search, and the exponential dependence of the number of LAM points on the number of independent degrees of freedom has a large impact on overall computational cost.

### 2.2. Formulation of LAMs with torsional groups

In the proposed approach, the vector of independent degrees of freedom θ is further partitioned into *N*_*G*_ subvectors θ_*g*_, *g* = 1, …, *N*_*G*_, referred to as torsional groups, such that each torsion belongs to exactly one group. Each such group is assumed to have an additive effect on the deviations of the intramolecular energy, the values of the dependent degrees of freedom and the point charges from their corresponding values *in vacuo* molecular conformation. Consequently, the deviation in any one of these quantities brought about by varying torsion angles in two different groups can be computed by summing the deviations incurred when the torsion angles in each group are varied independently.

To apply the above principle during the global search stage of the CSP algorithm, we need to derive a separate set of LAMs at a separate set of reference points for each torsional group *g* while keeping all other torsional groups *g*′ ≠ *g* at their *in vacuo* values.

More specifically, for each torsional group, *g* = 1, …, *N*_*G*_, we perform the following steps:

(1) We select a set of *N*_*g*, ref_ reference points, 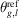, *l* = 1, …, *N*_*g*, ref_. These are completely independent of the reference points for any other torsional group.

(2) At each reference point *l* = 1, …, *N*_*g*,ref_, we perform an isolated-molecule QM conformational energy minimization:
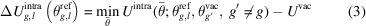
with respect to the dependent degrees of freedom 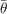 while fixing the independent degrees of freedom θ as follows:

(*a*) for group *g*, at the corresponding reference values 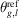


(*b*) for all other groups, *g*′ = 1, …, *N*_*G*_,  *g*′ ≠ *g*, at the corresponding *in vacuo* conformational values 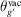.

(3) From the solution of minimization problem (3), we

(*a*) obtain the values of the dependent degrees of freedom, 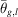


(*b*) determine the corresponding point charges *q*_*g*,*l*_.

(4) By analogy to (3), (6) and (8), we construct the following LAMs for the deviations of intramolecular energy, dependent degrees of freedom and point charges from the corresponding *in vacuo* values 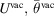 and *q*^vac^, respectively:
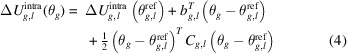

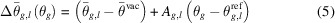

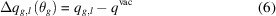
where we have introduced the vector:
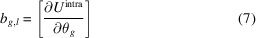
and the matrices:
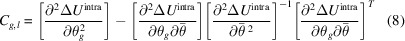

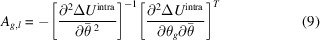
where all partial derivatives are computed at the solution of the minimization problem (3).

Overall, the above algorithm requires 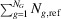 QM energy minimizations at step (2), plus one more for the initial (and subsequent if lower energy conformations are found) *in vacuo* conformation(s) [see equation (1)], and results in the construction and storage of 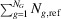 LAMs at step (4). By comparison, without the additivity principle, we would need 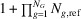 QM energy minimizations, which would be a significantly higher number for many practical problems.

Once constructed, the LAMs can be used during the global search stage to evaluate the corresponding quantities at any given set of values of the independent degrees of freedom, θ, based on the following algorithm:

Given the vector θ:

(1) For each torsion group *g* = 1, …, *N*_*G*_

  (*a*) determine the values of the corresponding subvector θ_*g*_

  (*b*) determine the reference point θ_*g*,*l*_ that is nearest to θ_*g*_

  (*c*) evaluate expressions (4)–(6) to determine, 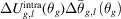, **Δ***q*_*g*,*l*_(θ_*g*_).

**Figure 1:**
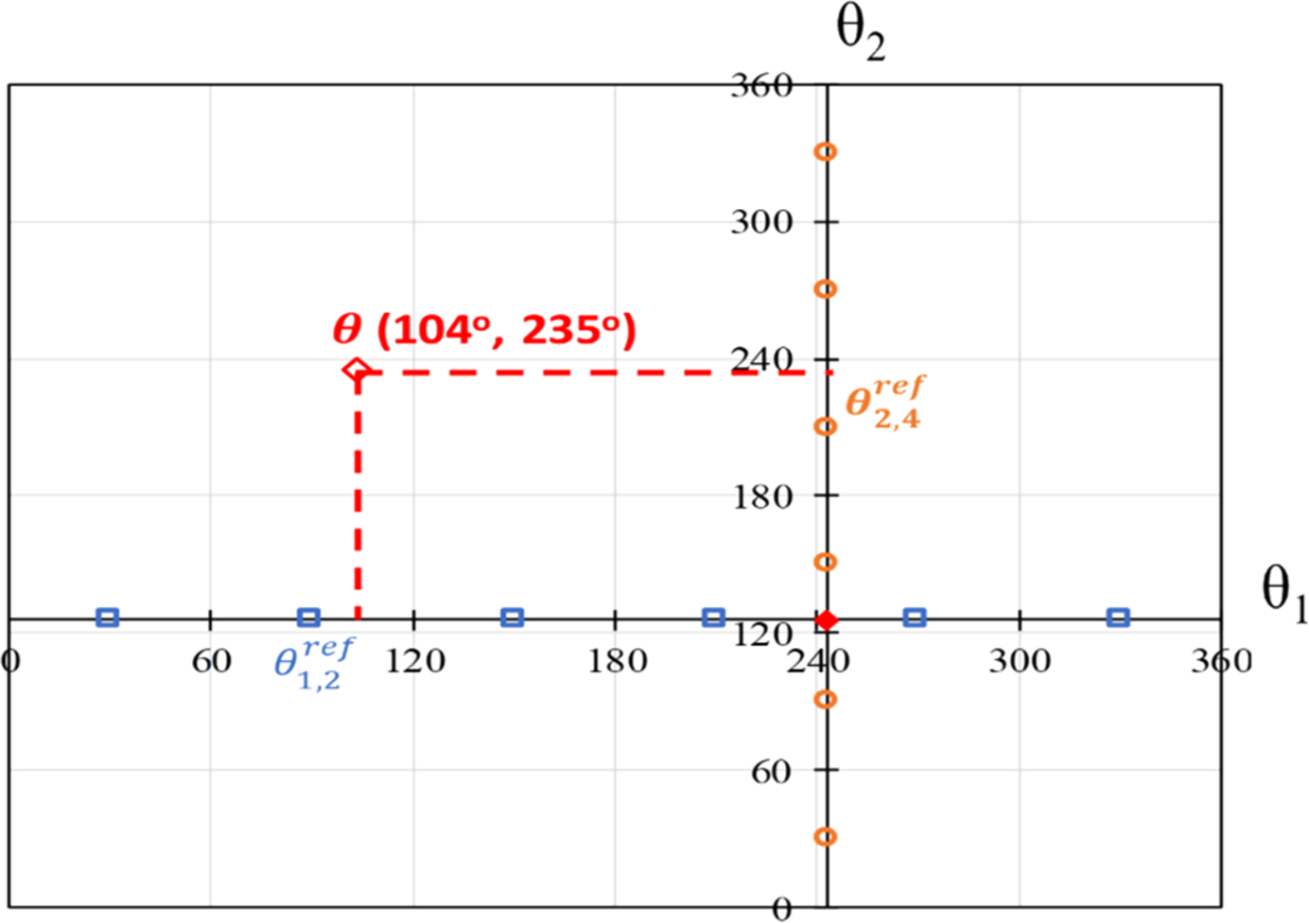
Illustration of the generation of reference points using the torsional group partitioning scheme. Two groups are considered, each with one independent degree of freedom: θ_1_ (blue) for group 1 and θ_2_ (orange) for group 2. The *in vacuo* minimum is shown in a filled red diamond at the intersection of the axes. The projected reference points are shown as blue squares for group 1 and orange triangles for group 2. The reference points in the two groups that would be used in the LAMs that are relevant to a particular point θ = (104°, 235°)^*T*^ in the space of the independent conformational degrees of freedom (see text for details) are shown. Torsions are given in °.

(2) Apply the additivity principle to determine the required values of the intramolecular energy, the dependent degrees of freedom and the point charges:
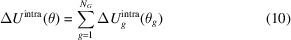

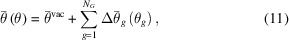

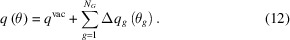
As an illustration, Fig. 1 ▸ considers a molecule with two independent degrees of freedom, θ_1_ and θ_2_, that have been trivially separated into two (*N*_*G*_ = 2) groups comprising one angle each; torsional ranges are given in the range 0–360° for sequencing clarity. The *in vacuo* values, 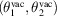 = (243°, 126°), are indicated by a red symbol. A uniform spacing of 60° is chosen to specify six reference points per group (*N*_ref, 1_ = *N*_ref, 2_ = 6) located at 30°, 90°, 150°, 210°, 270° and 330°, respectively, as indicated by the open squares and circles. Overall, before embarking on the global search, we would need to perform 13 QM energy minimizations, corresponding to these 12 reference points plus one more for the *in vacuo* conformation. We would then store the 13 sets of LAMs in terms of the corresponding quantities 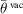, *q*^vac^, 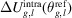, 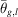, *q*_*g*,*l*_, *b*_*g*,*l*_, *C*_*g*,*l*_,*A*_*g*,*l*_ [*cf*. equations (4)–(9)].

Now consider a situation during the global search stage where we need to evaluate the lattice energy for a crystal structure involving a molecular conformation with θ = (104°, 235°)^T^. The closest reference points are 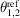 = 90° in the first torsional group and 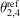 = 210° in the second one. The values of interest can be computed by evaluating the lower-dimensional LAMs generated at these points using equations (10), (11) and (12), and then combining these using equations in supporting information.

Finally, we note that the proposed approach does not require the reference points to be uniformly spaced. Therefore, these points can be generated by running an adaptive LAM generation algorithm (Sugden *et al.*, 2016 ▸) to convergence in each group *g* independently, with all flexible torsions belonging to other groups *g*′ ≠ *g* held at their *in vacuo* values.

### 2.3. Impact of partitioning of torsional groups on LAM accuracy

#### 2.3.1. Accuracy of LAMs for methyl paraben

**Figure 2:**
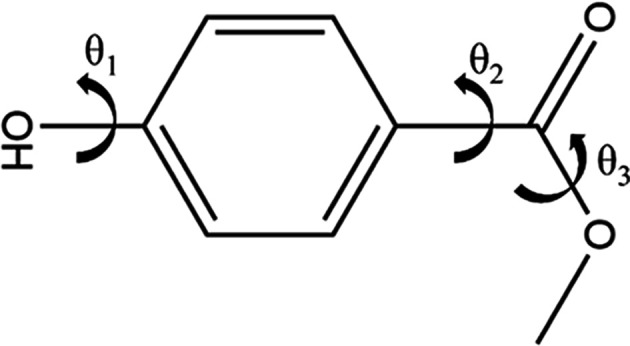
Molecular diagram of methyl paraben, with flexible torsions being indicated by curved arrows, and set at arbitrary values in this 2D diagram.

The torsional group partitioning algorithm is illustrated on methyl paraben, a small molecule from the GRAS list (Burdock & Carabin, 2004 ▸) with a significant degree of separation between the independent degrees of freedom, as can be seen in Fig. 2 ▸. Because the atoms in the hydroxyl and ester groups are in *para* positions on the central benzene ring, it can be expected that the effect of changes in the value of θ_1_ (hydroxyl) on the conformationally dependent variables (Δ*U*^intra^, point charges, and dependent degrees of freedom) will be nearly independent of the effect of any concurrent changes in θ_2_ or θ_3_ (ester). The same approximation cannot be applied to θ_2_ and θ_3_ however, since changes in the value of θ_2_  will have a direct impact on the strains involved in changing the value of θ_3_. Thus, two torsional groups are defined, *G*_1_ = {1} and *G*_2_ = {2, 3}.

To construct the LAM databases, reference points are generated using a uniform spacing of 60° for all independent degrees of freedom across the range (0°, 360°), with the first grid point at 30°; therefore, there are six points in each direction. Two LAM databases are then generated:

(1) LAM database without torsional group partitioning: in this case, there are a total of 6 × 6 × 6 = 216 reference points; 217 QM calculations are required, including the *in vacuo* calculation.

(2) LAM database with torsional group partitioning: in this case, there are six reference points for *G*_1_ and 6 × 6 = 36 reference points for *G*_2_, giving a total of 43 QM calculations, including the *in vacuo* calculation.

Overall, we note that the introduction of partitioning reduces the number of QM calculations by approximately 80%. However, the key question is whether this has a materially adverse effect on the accuracy of the predictions based on these LAMs. We assess this via the following procedure.

(1) We generate 2000 random points from the uniform probability distribution over the space of the flexible torsions and evaluate the intramolecular energy Δ*U*^intra^ at each point by performing a QM calculation [*cf*. equation (1)].

(2) We discard all points that are found to be outside the region of crystallographic relevance *i.e.* where Δ*U*^intra^ > 20.0 kJ mol^−1^. This leaves 110 points.

(3) For these 110 crystallographically relevant points, we evaluate the LAMs for both the partitioned and non-partitioned schemes and compare the results with the corresponding exact values. The average errors are given in Table 1 ▸. We note that both LAM generation schemes result in very similar prediction errors. Moreover, the latter are remarkably low considering that the LAMs were generated on a uniform grid with only six points for each torsional angle.

Additionally, Fig. S2 shows a parity plot of the Δ*U*^intra^ values predicted with the partitioned scheme at the 216 reference points used in the non-partitioned scheme. The *R*^2^ value is 0.9985, indicating that the hypothesis that there is very little interaction across the two torsional groups is justified.

Overall, it can be concluded that, for the case of methyl paraben, the approximation introduced by the additivity assumption does not lead to any significant loss in the quality of the model.

#### 2.3.2. Accuracy of LAMs for molecule XX

**Figure 3:**
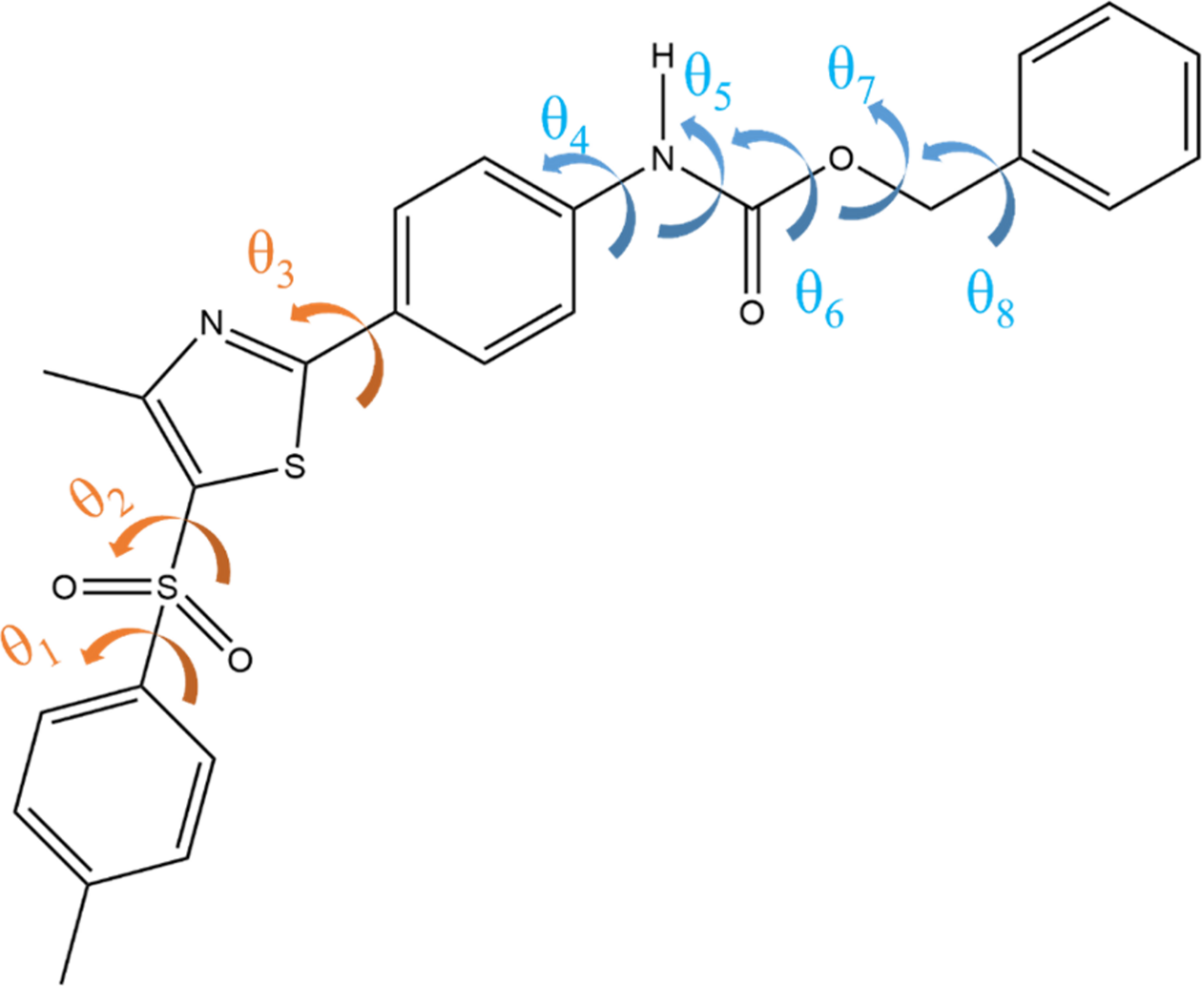
Molecular diagram of molecule XX, with flexible torsions indicated by arrows. Torsions 1–3 (orange) are assigned to torsional group 1, and 4–8 (blue) to torsional group 2.

A further assessment of the validity of the partitioning approximation is made by considering a much larger compound with greater flexibility, namely molecule XX from the fifth blind test (Bardwell *et al.*, 2011 ▸). As shown in Fig. 3 ▸, there are eight independent degrees of freedom, θ_1_ to θ_8_, with θ_6_ expected to vary only slightly around 180°. This set of torsions is partitioned into two groups by taking advantage of the physical separation arising from the presence of the central benzyl ring: *G*_1_ = {1, 2, 3} and *G*_2_ = {4, 5, 6, 7, 8}. Group *G*_1_ could conceivably be further partitioned into two separate groups, containing θ_1_ and θ_2_ on the one hand, and θ_3_ on the other hand. However, this is unnecessary as a group containing three torsions is computationally manageable.

For this compound, it is computationally intractable to cover the entire range of flexibility of the eight independent degrees of freedom with a non-partitioned LAM generation scheme. Therefore, for the purposes of comparison with the partitioned scheme, a narrower range of flexibility that extends by ±30° around a point neighbouring the experimental values of the independent degrees of freedom is considered. The selected ranges are shown in Table 2 ▸. A uniform grid is set up with 30° increments in all independent degrees of freedom, with the reference points positioned away from the edges of the grid (+15° from the lower bound and −15° from the upper bound), so that two points are needed in each direction. For the purpose of investigating LAM accuracy and in view of the limited flexibility of θ_6_, only a single grid point (at 180°) is employed for this torsion angle. This constraint is lifted during the CSP study of this molecule presented in Section 3.3.

Overall, the above approach leads to a manageable number of QM calculations for both LAM generation schemes:

(*a*) non-partitioned scheme: 2^7^ reference points, giving a total of 129 QM calculations (including the one for the *in vacuo* conformation);

(*b*) partitioned scheme: 2^3^ = 8 reference points for *G*_1_ and 2^4^ = 16 points for *G*_2_, giving a total of 25 QM calculations (including the one for the *in vacuo* conformation).

Overall, even with the significantly reduced ranges of torsional angle flexibility, the use of the partitioning scheme results in an approximately 80% reduction in the number of QM calculations. The remaining computational parameters are given in the supporting information.

To investigate the limits of the partitioning scheme, we also consider an extreme case where we partition the independent degrees of freedom into eight groups, each containing just one torsion. Once again, two reference points are used for each torsion, other than for θ_6_ for which only one is used, yielding a total of only 16 QM calculations (including the one for the *in vacuo* conformation).

**Figure 4:**
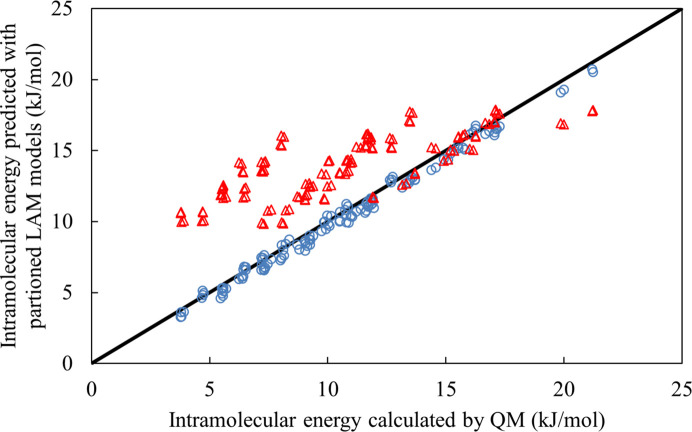
Parity plot for the intramolecular energy predicted by the LAM with torsional group partitioning versus intramolecular energy computed quantum mechanically for molecule XX. The blue circles correspond to the partitioning of the eight flexible torsions into two groups and the red triangles to full partitioning into eight independent groups.

Three lattice energy minimizations are then performed, respectively using the non-partitioned, two-group and eight-group LAM databases; all three minimizations use the experimental values of the independent degrees of freedom as the initial point. The RMSD_15_ between the minimum-energy crystal structure produced by the non-partitioned scheme and those resulting from the two-group and eight-group schemes are 0.0710 Å and 0.219 Å, respectively. This indicates that the partitioning into two groups has a very small impact on the resulting crystal structure. However, completely ignoring all interactions between adjacent torsions leads to an unacceptable change in accuracy. This is further confirmed by examining how the two partitioned databases perform at approximating Δ*U*^intra^ at the 129 QM-calculated reference points in the non-partitioned LAM database, as shown in Fig. 4 ▸. It can be seen that a high degree of accuracy is achieved in the two-group case [0.47 kJ mol^−1^ average absolute deviation (AAD) and 1.1 kJ mol^−1^ maximum error] while the eight-group partitioning exhibits a significant decrease in accuracy (3.55 kJ mol^−1^ AAD and 8.0 kJ mol^−1^ maximum error).

## 3. Application of torsional group partitioning to CSP

The proposed partitioning scheme has been implemented within the *CrystalPredictor II* software (Habgood *et al.*, 2015 ▸) for structure generation and global search. In this section, we investigate the effect of partitioning on CSP studies of four molecules: paracetamol, methyl paraben, molecule XX and safinamide. Following the approach highlighted in Pantelides *et al.* (2014 ▸), our CSP studies include a global search step followed by one refinement step to ascertain whether the experimental forms are found as low-lying energy minima. Further refinements to improve the reliability of the relative energy rankings are possible (Bowskill *et al.*, 2021 ▸) [as reported, for example in the sixth blind test (Reilly *et al.*, 2016 ▸) and in a recent study of the ROY molecule (Beran *et al.*, 2022 ▸)], but these are not undertaken here. Our focus is on examining whether the partitioning into torsional groups leads to a model that is sufficiently accurate to generate a global search landscape that leads to the successful identification of experimentally known polymorphs upon further refinement. Where the original non-partitioned scheme is computationally tractable, we also consider the relative reduction in computational cost achieved via partitioning.

### 3.1. Paracetamol

**Figure 5:**
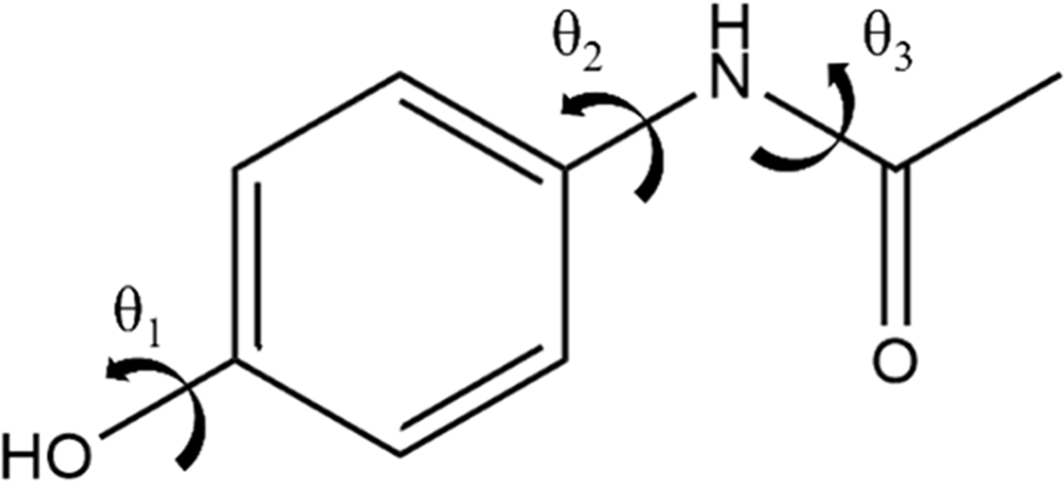
Molecular diagram of paracetamol, with flexible torsions indicated by arrows.

The new methodology is first applied to a small flexible active pharmaceutical ingredient (API), paracetamol (see Fig. 5 ▸). The three flexible torsions are separated geometrically by a *para* arrangement in a benzene ring, with θ_1_ on one side of the ring, and θ_2_ and θ_3_ on the other. This suggests that the flexible degrees of freedom can be partitioned into two groups: *G*_1_ = {1} and *G*_2_ = {2, 3}.

Two parallel investigations are performed without and with partitioning, in both cases using a uniform grid as summarized in Table 3 ▸, that covers a narrow range of conformational space around the known experimental conformation, as a proof of concept. The total number of QM calculations are 76 = 3 × 5 × 5 + 1 and 29 = 3 + 5 × 5 + 1, respectively. Thus, partitioning results in a 62% decrease in the number of these calculations.

**Figure 6:**
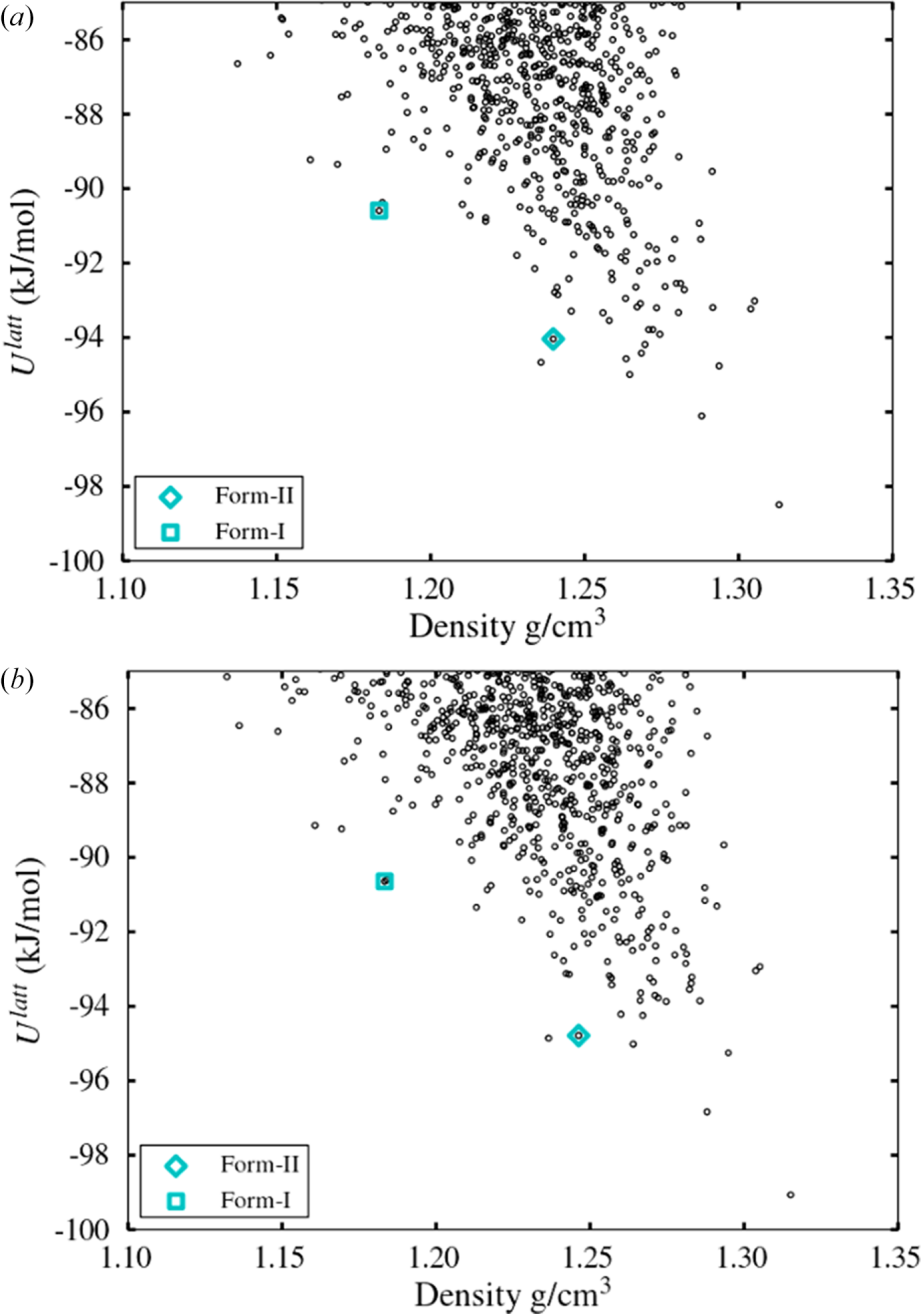
Polymorphic landscapes (lattice energy *U*^latt^ versus density of computed structures) after global search stage with *CrystalPredictor*, for paracetamol, in the (*a*) non-partitioned and (*b*) partitioned schemes.

Following the global search stage, the polymorphic landscape for the non-partitioned and partitioned schemes can be compared in Fig. 6 ▸. The main consideration at this stage is that the landscape should include structures that converge to the experimental crystal structures upon subsequent refinement with the more accurate, but also more expensive, energy model used in *CrystalOptimizer* (Kazantsev *et al.*, 2010 ▸), and that these structures should be low enough to be selected for such refinement, *e.g.* within 20 kJ mol^−1^ of the global minimum or within the lowest 1000 structures. For this simple case, it is clear that the two schemes result in nearly identical landscapes that include the experimental forms well within the 20 kJ mol^−1^ cutoff. This can be seen in more detail in Table 4 ▸.

Following refinement with *CrystalOptimizer* of all structures within 20 kJ mol^−1^ of the global minimum from the global search, the landscape shown in Fig. S2 is obtained with the structures generated by the non-partitioned LAM scheme. Polymorph I is observed as the global minimum structure, with an RMSD_15_ of 0.1933 Å relative to the experimental structure, whilst polymorph II is seen at rank 9, 3.23 kJ mol^−1^ above the global minimum, with an RMSD_15_ of 0.2487 Å. The refined landscape obtained with the structures generated by the partitioned LAM scheme are practically identical. This illustrates that the global search stage simply needs to be of sufficient accuracy to provide the refinement stage with good starting points from which to generate an accurate and complete list of viable structures. This is evidently achieved with both partitioning schemes.

The computational costs of the two approaches are compared in Table 5 ▸. A reduction of 63% is observed in the generation of the LAM database with the new scheme, in line with the reduction in the number of QM calculations. However, as LAM generation is relatively inexpensive for this small system, the overall CSP costs remain similar.

### 3.2. Methyl paraben

The next CSP study is conducted on methyl paraben (*cf*. Section 2.3.1), a compound with three known polymorphs. It is of a similar size to paracetamol, but we now consider the use of the adaptive LAM generation algorithm within *CrystalPredictor II* (Sugden *et al.*, 2016 ▸), as this is more representative of the approach one would typically take when investigating a new compound. By concentrating the reference points in those regions of conformational space that are of crystallographic relevance, adaptive LAM generation can greatly reduce the number of QM calculations required to study a molecule compared to a uniform grid of the same accuracy. Moreover, the adaptive algorithm can readily be applied to a partitioned set of degrees of freedom to reduce computational effort still further.

In the non-partitioned case, starting from a uniform grid with grid increments of 120.0° (*i.e.* 28 reference points), convergence of the adaptive LAM generation algorithm is reached after a total of 46 LAM points have been evaluated (Δ* = 5 kJ mol^−1^, Δ** = 20 kJ mol^−1^). Using the same grid increments, a partitioned LAM scheme based on the partitioning introduced in Section 2.3.1 initially contains three and nine LAM points in groups 1 and 2, respectively. The adaptive LAM generation algorithm is run independently for each group, and convergence is reached after a total of six and 23 reference points are generated in groups 1 and 2, respectively. Therefore, including the *in vacuo* configuration, there are 47 reference points in the non-partitioned case and 30 in the partitioned case. Thus, partitioning results in a 36% decrease in the number of QM calculations.

**Figure 7:**
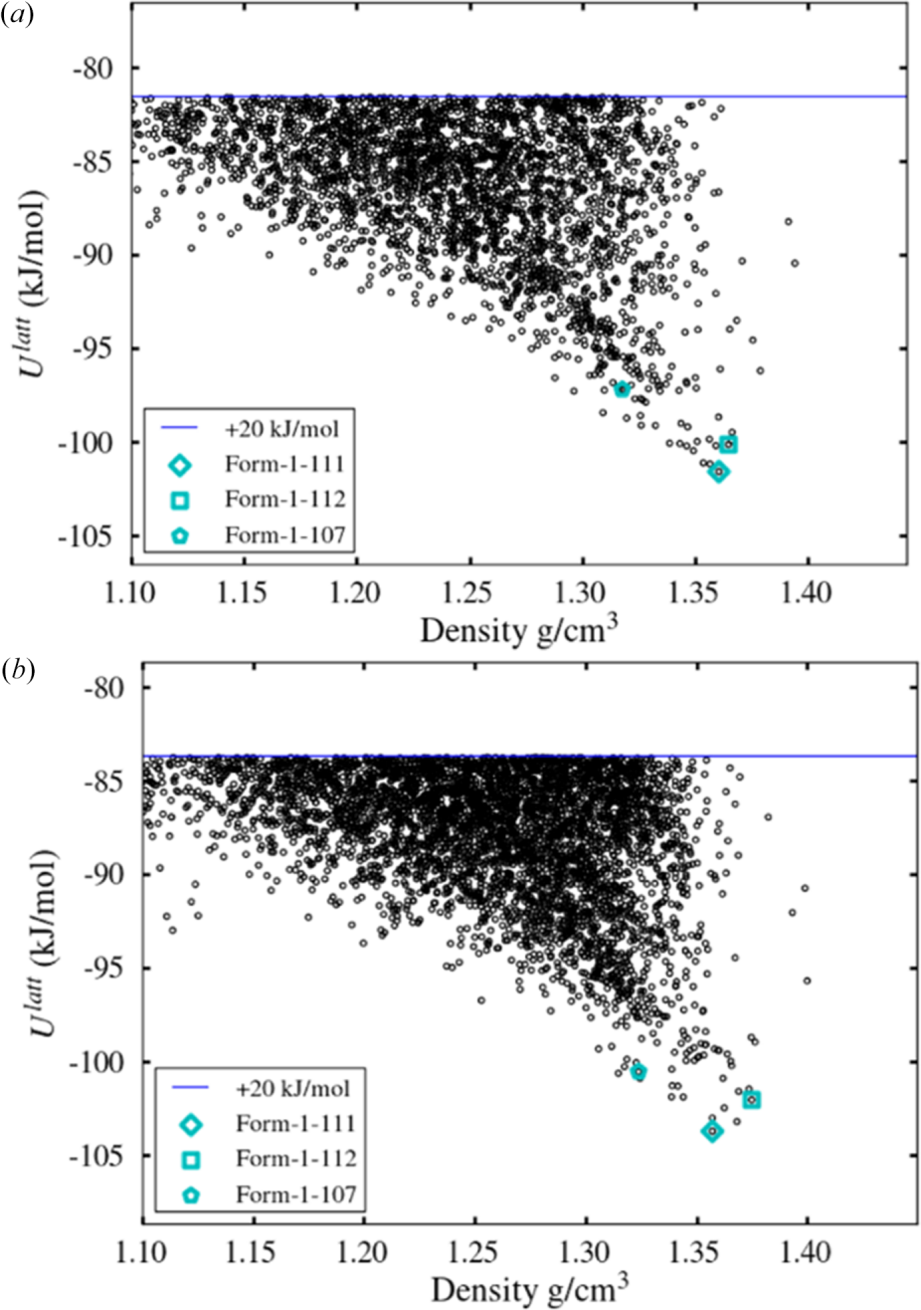
Polymorphic landscapes for methyl paraben within 20 kJ mol^−1^ of the global minimum after global search stage with *CrystalPredictor II* in the (*a*) non-partitioned and (*b*) partitioned schemes.

Following the global search stage, all three experimental polymorphs are found by both LAM schemes within 5 kJ mol^−1^ of the global minimum (see Fig. 7 ▸) with an accurate geometrical representation of the experimentally observed polymorphs, as indicated by the RMSD_15_ values listed in Table 4 ▸. The impact of partitioning on CPU time is shown in Table 5 ▸. As in the case of paracetamol, the use of partitioning results in a significant reduction in the CPU time required for LAM generation, but the effect on the total CPU time is small.

### 3.3. Molecule XX

As the two molecules considered in Sections 3.1 and 3.2 had only three independent conformational degrees of freedom, the corresponding CSP studies were tractable even without the use of the partitioning scheme presented in this paper. In this section, we consider the application of the partitioning scheme to molecule XX of the fifth blind test (*cf*. Section 2.3.2) which has eight flexible torsion angles. In this case, a CSP study using a standard non-partitioned LAM scheme would be computationally intractable.

Successful CSPs of this molecule were carried out in the fifth blind test (Bardwell *et al.*, 2011 ▸; Kazantsev, Karamertzanis, Adjiman, Pantelides *et al.*, 2011 ▸), including one by our research group using *CrystalPredictor I*. As has already been mentioned, the latter uses restricted Hermite interpolants instead of LAMs, which practically limits the number of independent torsions that can be handled within a single torsional group to three. To address this limitation, torsion θ_4_ was fixed at 0° and the flexibility in θ_5_ was approximated by fixing this torsion to two distinct values, namely either 0 or 180° (*cis* or *trans* configurations) and performing separate searches for each option. Further, the restricted Hermite interpolant scheme necessitates a uniform grid, requiring a large number of QM calculations. To make this tractable, the computational cost was reduced by using a combination of one-dimensional scans and Crystal Structure Database analysis to identify the most likely ranges of values of the flexible torsions and the corresponding energetically meaningful regions of conformational space. A separate search was then performed for each such region. In total, this led to eight separate searches. The group of Professor Graeme Day at the University of Southampton was also successful in predicting the experimental form of molecule XX by conducting multiple (48) rigid-molecule searches to identify initial structures (Price *et al.*, 2010 ▸).

However, while the use of separate global searches proved successful in this particular case, the assumptions made to narrow the search space cannot generally be relied upon. In contrast, the partitioning approach presented in this paper combined with the adaptive LAM generation algorithm allows for full coverage of conformational space within a single global search. The LAM generation is initiated with a uniform grid for each torsional group (see Section 2.3.2) with increments of 120°, except for θ_5_, for which an increment of 180° is used, as shown in Table 6 ▸. The subsequent application of the adaptive LAM generation algorithm results in 8461 LAMs for group 1 and 334 LAMs for group 2, *i.e.* a total number of 8796 QM calculations (including the one required for establishing the *in vacuo* conformation). A non-partitioned database of equivalent accuracy would require more than 2.8 million (∼8461 × 334) QM calculations, which would be prohibitively expensive even with modern computer resources.

**Figure 8:**
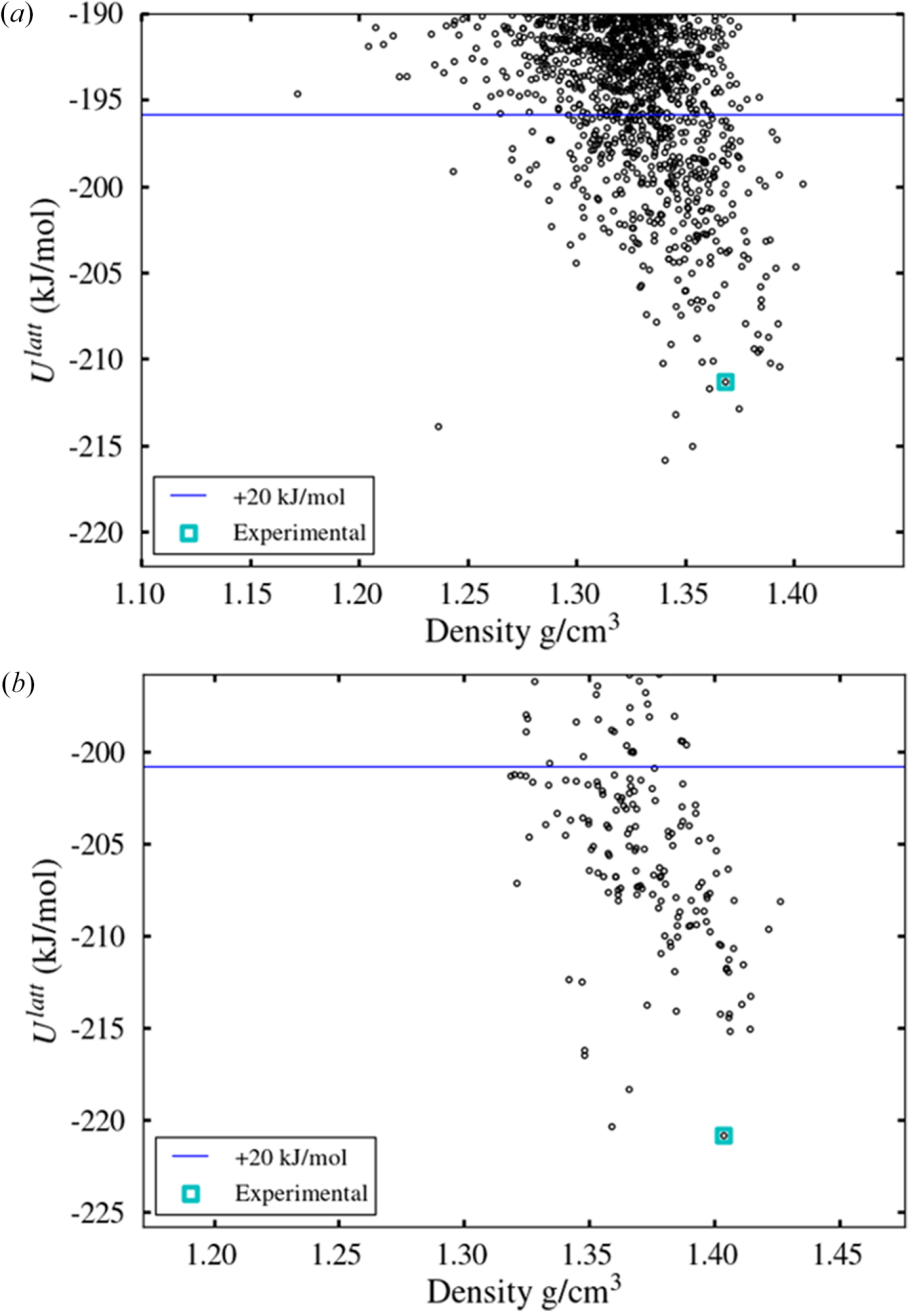
Polymorphic landscape for molecule XX, after (*a*) global search and (*b*) refinement.

The global search landscape is shown in Fig. 8 ▸(*a*). The experimental form is at rank 9 and 4.54 kJ mol^−1^ above the global minimum. This indicates that the energetic representation provided by the model is sound, whilst the RMSD_15_ of 0.378 Å confirms the quality of the geometric representation.

Finally, the 411 structures that are found to be within the usual 20 kJ mol^−1^ cutoff are refined with *CrystalOptimizer* using the same level of theory as in the original study (Kazantsev, Karamertzanis, Adjiman, Pantelides *et al.*, 2011 ▸). The polymorphic landscape is seen in Fig. 8 ▸(*b*) where the experimental form is, once again, observed as the global minimum. The RMSD_15_ comparison between the experimentally observed and predicted structure is 0.186 Å. Overall, these results confirm the reliability of the proposed partitioning scheme.

The CPU cost of the CSP study is reported in Table 7 ▸. As can be seen, the dominant component is now the LAM database generation and the savings afforded by the partitioning are therefore critical to the success of the study. We note that the overall cost shown here is larger than that of the original study. This is in large part due to the fact that the full search space is explored in our current work, whereas only a subset of the conformational space was investigated previously.

### 3.4. Safinamide

**Figure 9:**
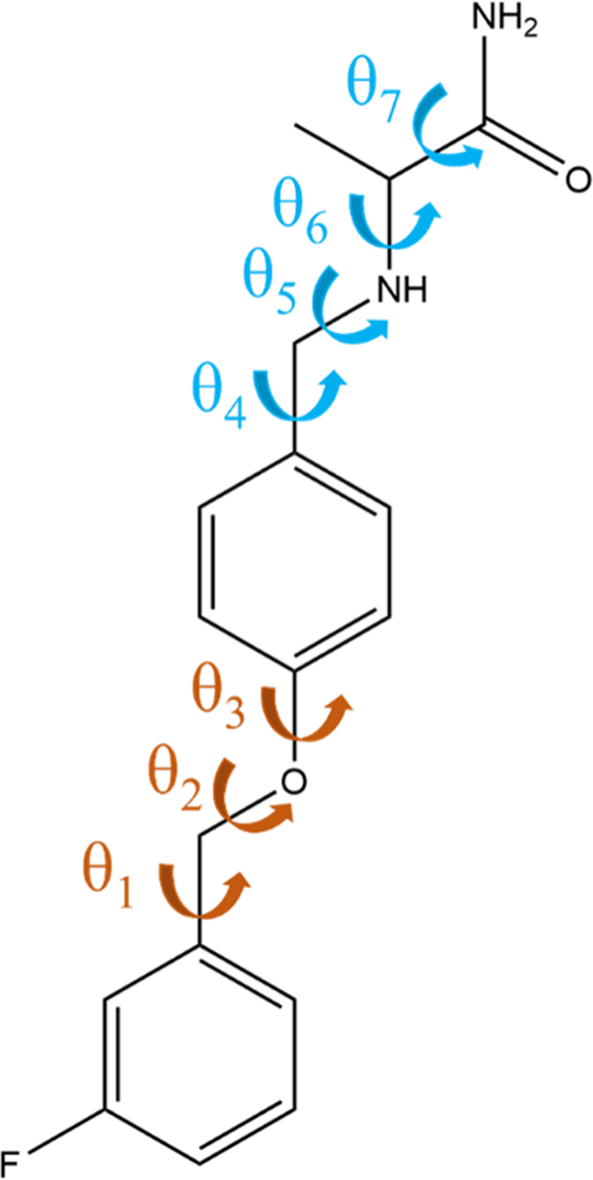
Molecular diagram of safinamide, with flexible degrees of freedom indicated. Group 1 (θ_1_–θ_3_) degrees of freedom are shown in orange, whilst group 2 (θ_4_–θ_7_) is shown in blue.

As a final assessment of the performance of the proposed approach, we carry out a CSP study of safinamide, a drug that is used to treat Parkinson’s disease (Leuratti *et al.*, 2013 ▸). To our knowledge, this is the first time that such a study is being reported for this molecule in the open literature. The safinamide molecule involves 41 atoms and seven torsional degrees of freedom, as seen in Fig. 9 ▸. It has two known conformational polymorphs (Ravikumar & Sridhar, 2010 ▸; Cruz-Cabeza & Bernstein, 2014 ▸). Form I (CSD refcode TUWFIB) features an unusual amine N—H⋯F contact, whilst in form II (TUWFIB01) the hydrogen bonding scheme satisfies all of the NH donors/acceptors, which is indicative of stability ranking This example represents a sighted test, in that experimental polymorphs are known before the CSP investigation, but illustrates the application for a given molecule before a polymorphic screen.

The independent degrees of freedom in the safinamide molecule are geometrically separated either side of a benzene moiety, making it an ideal, if challenging, test case for the proposed algorithm. More specifically, we employ two torsional groups: group 1 with torsions θ_1_ to θ_3_, and group 2 with torsions θ_4_ to θ_7_, assuming complete flexibility in all cases. Starting from a uniform grid with three increments of 120° spanning 360° degrees in each torsion and applying the adaptive LAM algorithm until convergence, we construct LAM sets containing 1148 and 4121 reference points for groups 1 and 2, respectively. An equivalent non-partitioned database would require approximately 4.7 million LAMs.

**Figure 10:**
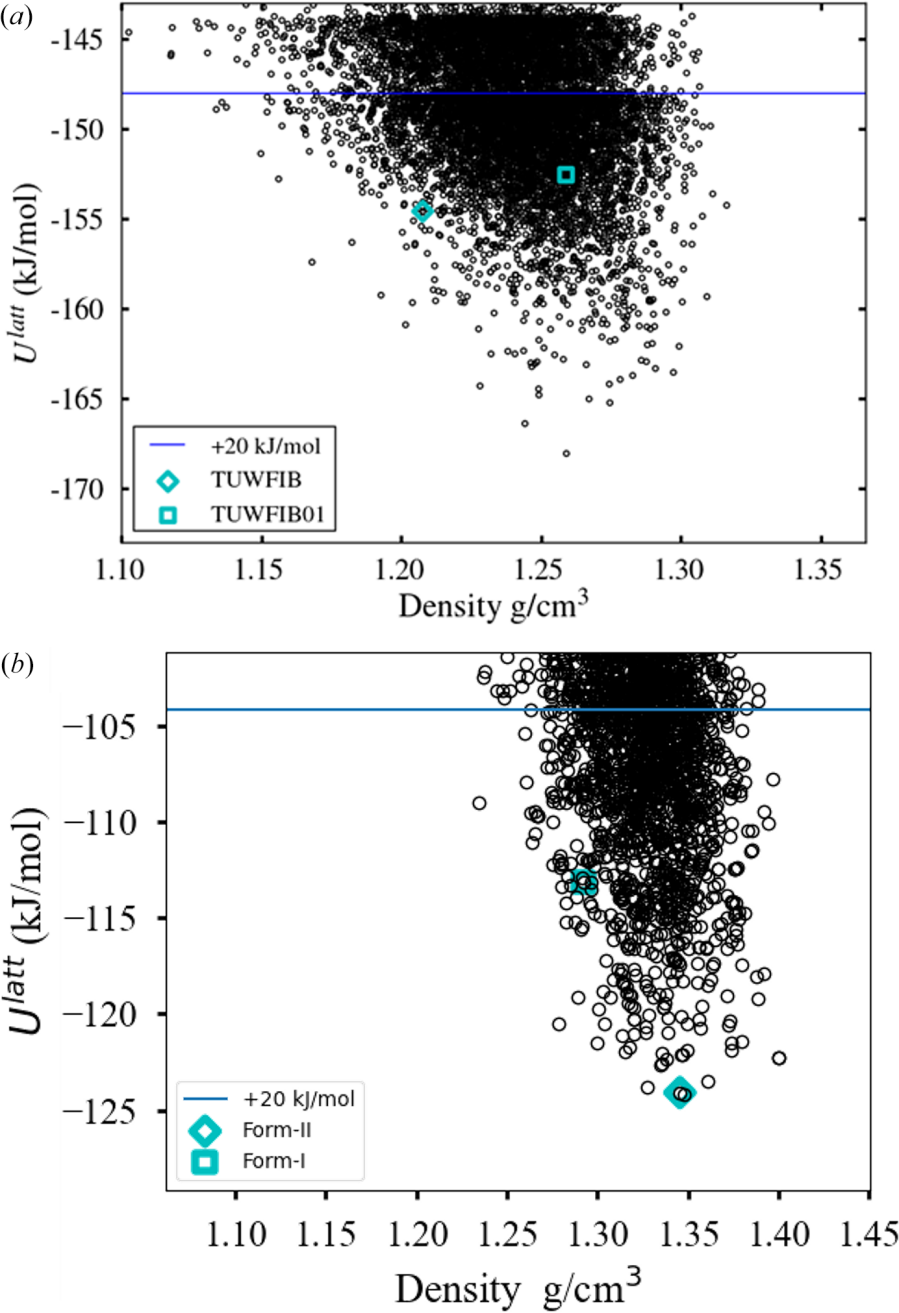
(*a*) Safinamide polymorphic landscape after global search with partitioned LAMs. (*b*) Safinamide polymorphic landscape after refinement of the 3000 lowest-energy structures identified by the global search.

The polymorphic landscape after a global search of 500000 minimizations is shown in Fig. 10 ▸. A structure matching form I is found 13.5 kJ mol^−1^ above the global minimum at rank 572, and a structure matching form II is found at 15.5 kJ mol^−1^ above the global minimum at rank 1165. Although high in energy, both forms are well within the normal cutoff of 20 kJ mol^−1^, with good geometric representation: the RMSD_15_ for form I is 0.355 Å, and that for form II is 0.347 Å. There are 8250 structures within 20 kJ mol^−1^ of the global minimum in total.

Following refinement of the 3000 lowest-energy structures, form I is observed at rank 150, 10.97 kJ mol^−1^ above the global minimum, whilst form II is commensurate with the global minimum (+0.08 kJ mol^−1^, rank 2), as seen in Fig. 10 ▸(*b*). The geometric representation is satisfactory for both forms, with an RMSD_15_ for form II of 0.2232 Å, and for form I of 0.4142 Å. This provides some confidence that the two structures will be identified as low-lying minima upon further refinement using even more accurate lattice energy models, such as those computed via periodic DFT calculations (Hafner & Kresse, 1997 ▸). Once again, Table 8 ▸ shows the LAM generation to be a significant cost and the use of partitioning brings this down significantly, to be of the same order of magnitude as the first refinement step. The fact that these initial steps lead to the identification of the two experimental forms within the top 150 structures within less than 400000 CPU hours is encouraging for such a large molecule. Within a pharmaceutical context, an indication that an experimentally known form is commensurate with the thermodynamic global minimum would provide confidence in the completeness of the polymorphic screen.

## 4. Concluding remarks

We have presented an approach for the calculation of the lattice energy of putative crystal structures based on a partitioning of the molecule’s independent conformational degrees of freedom into torsional groups. The approach can significantly reduce the number of isolated molecule QM calculations required to conduct a global search of the potential crystal structures of organic molecules. It is applicable to molecules where there is sufficient geometric separation between the torsional groups. In such cases, the combined effects of the deviations of torsional angles belonging to different groups on the intramolecular energy, molecular conformation and electronic charge distribution are approximately additive.

The validity of the additivity approximation was demonstrated using systems which are computationally tractable even without the partitioning. These included two small molecules (paracetamol and methyl paraben) and a larger one (molecule XX from the fifth blind test) with restricted ranges of variation of the flexible torsions.

The partitioning approach was further tested on two systems of such size and flexibility that would otherwise be considered as practically intractable: molecule XX with the full ranges of variation of the flexible torsion angles, and safinamide. The global search landscapes generated in both cases were found to be of high quality, with all known experimental structures ranked low enough in energy to be considered for further refinement. Further work will include using the method on ever larger systems, with more rotatable torsions, to understand where further improvements to efficiency are required for practical applications

## 5. Related literature

The following references are cited in the supporting information for this article: Bowskill (2021 ▸), Frisch *et al.* (2016 ▸), Hu *et al.* (2007 ▸) and Williams & Cox (1984 ▸).

## Supplementary Material

A more thorough computational breakdown for each example, data for the methyl paraben comparison, and force-field parameters for the safinamide investigation. DOI: 10.1107/S2052520624010072/ra5144sup1.pdf

## Figures and Tables

**Table 1:** Average errors in the conformationally dependent properties of methyl paraben at 110 crystallographically relevant points using the non-partitioned and partitioned LAM schemes, relative to QM-calculated values

LAM generation scheme	Intramolecular energy (kJ mol^−1^)	Bond lengths (Å)	Bond angles (°)	Torsion angles (°)	Charge (e)
Non-partitioned	1.68	0.0004	0.09	0.5	0.002
Partitioned	1.39	0.0004	0.07	0.5	0.002

**Table 2:** Experimental values and range of torsion values used to assess the quality of the proposed approximation for molecule XX The grid midpoint is determined by choosing a value that includes the experimental value and the expected end point from minimizations using the standard LAM database, rounded to the nearest 10° angle. The upper and lower bounds are then set at ±30° of this midpoint and two reference points are introduced for each torsion, except for θ_6_, where the bounds are set at ±15° and one reference point is used.

Torsion	Experimental value (°)	Grid midpoint (°)
θ_1_	107.04	110
θ_2_	104.74	90
θ_3_	167.52	180
θ_4_	1.09	0
θ_5_	176.41	180
θ_6_	185.72	180
θ_7_	254.18	270
θ_8_	261.95	270

**Table 3:** Uniform LAM grid for investigation of paracetamol

Torsion	Grid lower bound (°)	Grid upper bound (°)	Grid increment (°)	Number of distinct torsion values
θ_1_	−30	30	±15	3
θ_2_	120	240	±15	5
θ_3_	120	240	±15	5

**Table 4:** Lattice energies, rank and RMSD_15_ for paracetamol and methyl paraben using the non-partitioned and partitioned schemes

	Non-partitioned LAM scheme			Partitioned LAM scheme		
Experimental form (CSD refcode)	*U*^latt^ (kJ mol^−1^)	Rank	RMSD_15_ (Å)	*U*^latt^ (kJ mol^−1^)	Rank	RMSD_15_ (Å)
Paracetamol						
Monoclinic polymorph I (HXACAN57)	7.90	77	0.398	8.42	91	0.406
Orthorhombic polymorph II (HXACAN08)	4.45	9	0.491	4.28	6	0.527

Methyl paraben						
1-111 (CEBGOF03)	–	1	0.251	–	1	0.215
1-112 (CEBGOF04)	1.45	5	0.218	1.68	5	0.306
1-107 (CEBGOF05)	4.38	15	0.110	3.18	17	0.205

**Table:** 

Approximate computational cost (CPU hour)		
Paracetamol		
Stage	Non-partitioned LAM scheme	Partitioned LAM scheme
LAM generation	390	145
Global search	2000	2000
Refinement	18000	18000

**Table:** 

Methyl paraben		
Stage	Non-partitioned LAM database	Partitioned LAM database
LAM database generation	100	65
Global search	1250	1250

**Table 6:** Description of partitioned LAM database for molecule XX

Torsional group	Torsion angle	Initial uniform grid			Number of adaptive LAM points
		[Lower bound, increment, upper bound ] (°)	Number of reference points	Number of LAM points	
1	θ_1_	[60, 120, 300]	3	27	307
	θ_2_	[−30, 120, 210]	3		
	θ_3_	[60, 120, 300]	3		
2	θ_4_	[−120, 120, 120]	3	162	8299
	θ_5_	[0, 180, 180]	2		
	θ_6_	[60, 120, 300]	3		
	θ_7_	[30, 120, 270]	3		
	θ_8_	[−30, 120, 210]	3		

**Table 7:** Approximate CPU cost of each stage of a CSP study for molecule XX, using a partitioned LAM database CPU cost equates to 3–6 months on standard HPC architecture.

Stage	Approximate computational cost (CPU hour)
LAM database generation	700000
*CrystalPredictor* global search	60000
*CrystalOptimizer* refinement	90000

**Table 8:** Approximate CPU cost of each stage of a CSP for safinamide, using a partitioned LAM database

Stage	Approximate computational cost (CPU hour)
LAM database generation	240000
*CrystalPredictor* global search	4000
CSO-FM refinement	140000

## Data Availability

All data accompanying this paper are available at https://zenodo.org/records/10433580.
