# Supplementary material for: Accurate and efficient representation of intramolecular energy in ab initio generation of crystal structures. Part III: partitioning into torsional groups
Source: Acta Crystallogr B Struct Sci Cryst Eng Mater. 2025 Jan 22;81(Pt 1):114–27. doi: 10.1107/S2052520624010072 (PMC11801705; doi:10.1107/S2052520624010072)
Supplement: Supplementary file 1 [file b-81-00114-sup1.pdf]

STRUCTURAL SCIENCE
CRYSTAL ENGINEERING
MATERIALS

Volume 80 (2024)

Supporting information for article:

Accurate and efficient representation of intramolecular energy in *ab initio* generation of crystal structures. Part III: partitioning into torsional groups

Isaac J. Sugden, David H. Bowskill, Benjamin I. Tan, Yizu Zhang, Claire S. Adjiman and Constantinos C. Pantelides

Equations to support the main text

Intramolecular energy difference, ΔU^intra:

$$\Delta U^{intra}(\boldsymbol{\theta}) = \Delta U^{intra}\left(\boldsymbol{\theta}_l^{ref}\right) + \boldsymbol{b}_l^T\left(\boldsymbol{\theta} - \boldsymbol{\theta}_l^{ref}\right) + \frac{1}{2}(\boldsymbol{\theta} - \boldsymbol{\theta}_l^{ref})^T \boldsymbol{C}_l\,(\boldsymbol{\theta} - \boldsymbol{\theta}_l^{ref}), \quad (1)$$

where $\boldsymbol{b_l}$ and $\boldsymbol{C_l}$ are, respectively, an L-dimensional vector and a $L \times L$ matrix evaluated at the solution of the minimization problem **Error! Reference source not found.** using the following expressions[8]:

$$\boldsymbol{b}_l = \left[\frac{\partial U^{intra}}{\partial \boldsymbol{\theta}}\right] \ , \quad (2)$$

$$\boldsymbol{C}_l = \left[\frac{\partial^2 \Delta U^{intra}}{\partial \boldsymbol{\theta}^2}\right] - \left[\frac{\partial^2 \Delta U^{intra}}{\partial \boldsymbol{\theta} \partial \overline{\boldsymbol{\theta}}}\right] \left[\frac{\partial^2 \Delta U^{intra}}{\partial \overline{\boldsymbol{\theta}}^2}\right]^{-1} \left[\frac{\partial^2 \Delta U^{intra}}{\partial \boldsymbol{\theta} \partial \overline{\boldsymbol{\theta}}}\right]^T . \quad (3)$$

(ii) Dependent conformational degrees of freedom, $\overline{\boldsymbol{\theta}}$:

$$\overline{\boldsymbol{\theta}}(\boldsymbol{\theta}) = \overline{\boldsymbol{\theta}}_l^{ref} + \boldsymbol{A}_l\left(\boldsymbol{\theta} - \boldsymbol{\theta}_l^{ref}\right), \quad (4)$$

where $\boldsymbol{A_l}$ is a $M \times L$ matrix evaluated at the reference point $\boldsymbol{\theta}_l^{ref}$ using the following expression[8]:

$$\boldsymbol{A}_l = -\left[\frac{\partial^2 \Delta U^{intra}}{\partial \overline{\boldsymbol{\theta}}^2}\right]^{-1} \left[\frac{\partial^2 \Delta U^{intra}}{\partial \boldsymbol{\theta} \partial \overline{\boldsymbol{\theta}}}\right]^T . \quad (5)$$

(iii) Point charges, $\boldsymbol{q}$:

$$\boldsymbol{q}(\boldsymbol{\theta}) = \boldsymbol{q}_l, \quad (6)$$

where $\boldsymbol{q_l}$ are the point charges derived from the QM electrostatic potential at the corresponding reference point, for example by using the HLYGAt approach[28].

Computational method

Unless otherwise stated, the molecules were first geometry minimised in the gas phase at the PBEPBE/6-31G(d,p) level of theory using Gaussian09[1]. Flexible torsions were determined through second derivatives and finite difference perturbations. The global search was performed using CrystalPredictor II[2], with the smoothed intramolecular potential algorithm[3], using 500,000 minimisations in each investigation. Dispersion–repulsion contributions towards the lattice energy were estimated by using a Buckingham exp-6 function with the potential parameters for Carbon, Hydrogen, polar Hydrogen, Sulphur and Oxygen from the FIT set[4]. The lattice energies reported in the landscapes are given per molecule.

Paracetamol – Section 3.1

Local approximate models (LAMs) were constructed by using a uniform grid along the Degrees Of Freedom (DOFs), at 30° increments. Following analysis and clustering, CrystalOptimizer[5] was used to refine the structures within 20 kJ/mol from the global minimum, at the same level of theory, with extra flexibility introduced (angles around previously flexible torsions).

Methyl Paraben

Information for Section 2.3.1

Local approximate models (LAMs) were constructed by using a uniform grid along the DOFs, at 60° increments

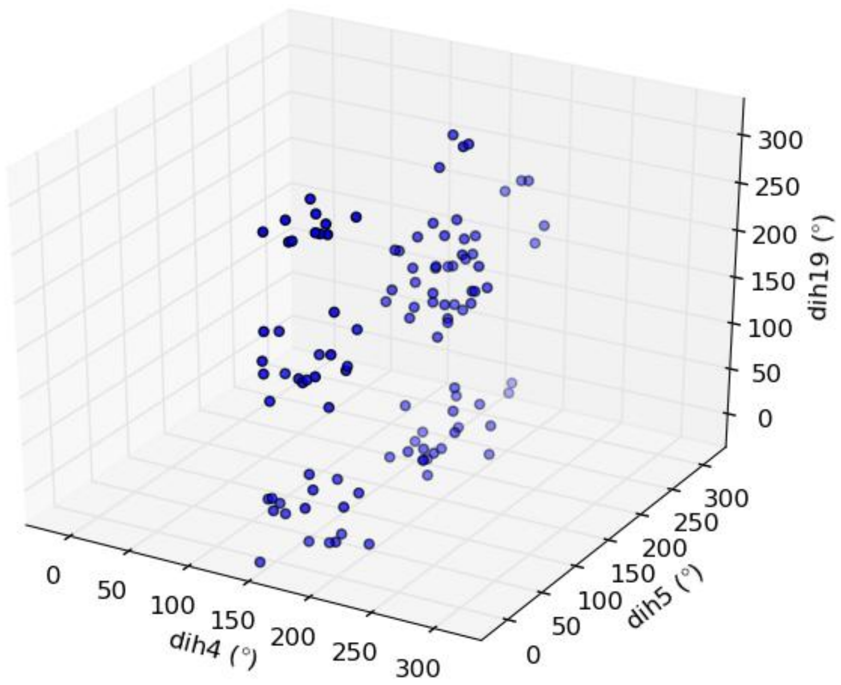

Figure 1. Distribution of 110 points considered in Section 2.3.1, that met the Uintra<20 kJ/mol cutoff. Tor1=dih19,Tor2=dih5,Tor3=dih4

Table 1. Points considered for methyl paraben in Section 2.3.1, with flexible torsion angles, and U_{intra} calculated using QM, CrystalPredictor with torsional group partitioning, and without torsional group partitioning

Point No.	Tor1 (°)	Tor2 (°)	Tor3 (°)	U_{intra} (kJ/mol)		
				QM	With partitioning	Without partitioning
15	203.28	171.71	216.46	16.41	15.05	14.68
29	188.07	228.17	22.61	20.56	19.74	19.61
63	174.86	5.70	61.87	18.17	17.82	18.04
88	159.56	145.22	-0.33	15.13	13.09	12.31
99	177.37	40.14	38.46	18.76	17.04	17.07
115	175.11	227.48	177.20	16.33	15.60	15.88
154	191.48	13.40	-10.03	3.25	3.34	2.62
161	184.43	221.12	188.10	12.84	11.80	11.76
172	204.46	169.10	329.14	14.93	13.35	12.90
182	169.58	162.54	156.34	7.27	6.88	5.85
256	199.02	30.65	321.09	19.32	16.64	15.62
303	207.12	-1.78	41.30	19.36	18.17	17.62
307	146.36	162.44	155.65	20.89	18.63	17.74
315	180.41	-16.26	175.50	1.90	1.11	1.40
318	193.56	196.51	316.53	14.75	14.80	13.52
326	159.05	-19.65	147.54	15.30	13.61	12.46
349	186.80	204.02	213.00	11.63	10.48	9.27
373	172.40	151.10	151.29	12.13	10.36	9.22
402	182.02	320.39	151.52	15.73	13.22	13.59
479	191.43	44.40	198.47	17.13	15.31	15.79
480	174.28	213.11	135.31	19.87	18.06	17.68
491	185.08	-6.41	55.10	15.50	15.18	15.35
520	208.12	177.69	176.80	10.62	9.80	9.14
526	166.96	187.97	-23.26	6.29	5.28	5.34
528	190.24	159.94	131.70	17.76	17.03	16.79
532	200.04	-9.06	322.92	14.00	12.65	12.59
550	216.78	184.28	179.66	17.05	16.01	15.65
551	166.32	224.38	168.80	17.76	15.98	16.67

552	205.26	36.51	-20.95	20.11	16.07	15.74
603	198.36	-24.15	187.03	9.66	7.73	8.27
608	169.33	167.33	227.84	15.64	15.83	15.02
616	204.11	183.78	159.71	10.65	9.77	8.97
622	203.51	217.15	9.30	18.91	16.26	15.82
672	158.96	-28.42	193.45	12.69	10.04	9.24
686	175.81	319.93	217.26	19.47	17.08	17.63
703	188.62	143.73	18.53	12.91	10.51	10.87
705	182.06	-13.73	170.63	1.95	1.10	1.54
717	208.85	185.96	199.05	13.69	12.42	11.61
729	150.67	7.90	203.16	15.98	14.36	14.15
760	152.14	327.92	195.99	19.20	14.46	14.08
772	156.00	188.73	-7.60	9.17	8.45	8.11
811	182.97	182.05	225.93	12.30	11.96	12.00
824	186.81	153.02	-16.13	7.73	6.08	5.88
825	171.21	27.07	175.38	6.46	4.41	4.89
830	152.36	-13.88	314.68	22.24	21.17	19.93
863	160.87	-28.90	224.48	22.22	19.77	19.00
879	182.92	131.05	20.65	19.62	18.89	18.88
883	153.03	205.23	-6.87	15.74	14.33	13.93
888	192.74	11.35	233.21	18.53	18.15	18.02
897	160.18	2.54	320.82	13.98	12.92	12.75
929	174.50	4.52	161.68	2.95	1.75	2.46
949	158.68	-0.34	17.73	8.01	7.34	6.88
972	193.70	149.79	12.18	10.64	8.10	8.53
986	203.07	-10.93	204.14	12.60	10.74	11.08
999	189.11	176.81	142.64	9.81	8.91	8.90
1035	164.72	-1.07	301.87	19.43	19.10	19.03
1056	157.12	172.07	41.30	17.03	16.35	15.36
1074	180.98	39.27	-19.46	12.29	10.55	10.64
1106	199.98	31.26	35.72	18.89	15.78	14.84
1120	189.13	172.12	159.70	4.26	3.57	3.57
1143	206.65	209.80	-17.63	18.50	15.69	14.04

1151	183.64	16.49	316.67	11.82	11.47	10.97
1161	152.66	-25.96	-22.25	17.19	14.23	12.74
1201	152.64	170.50	207.13	15.42	13.89	12.76
1209	165.75	166.96	194.41	5.20	5.30	4.33
1215	160.51	-19.49	32.31	13.40	12.30	10.76
1261	161.82	148.35	221.19	22.17	20.46	18.98
1265	177.06	28.89	-24.36	8.99	6.88	6.54
1282	177.49	215.34	0.43	9.30	7.58	7.58
1350	170.75	25.02	319.24	14.48	12.94	12.65
1354	176.17	177.43	239.26	17.96	17.84	17.85
1368	189.53	320.07	172.67	12.68	10.72	11.50
1385	162.82	24.39	-25.09	12.13	10.19	10.02
1417	207.15	156.56	35.45	22.03	20.12	19.46
1420	190.43	-26.20	48.35	18.32	17.01	16.77
1421	160.18	329.25	-14.84	12.76	10.32	9.41
1456	147.43	-4.96	32.50	19.98	17.95	17.33
1470	189.13	141.81	7.24	12.34	10.32	10.85
1473	155.57	-21.47	175.74	11.19	9.67	8.46
1475	177.13	184.28	293.82	19.55	19.41	19.49
1480	163.91	317.63	-19.03	17.53	15.53	15.65
1486	147.35	0.40	30.18	19.26	17.43	16.83
1494	185.52	205.32	136.55	16.00	15.43	14.05
1510	179.54	15.52	306.23	15.80	15.66	15.40
1511	187.05	192.30	238.83	19.34	19.48	19.00
1549	193.64	177.95	43.27	12.99	12.32	12.35
1580	174.10	208.83	315.26	17.31	15.62	15.13
1615	210.35	177.87	177.07	12.21	11.31	10.64
1634	186.00	174.78	1.33	0.77	0.51	0.57
1646	181.39	168.71	161.91	2.95	2.53	2.38
1650	165.26	328.49	210.63	15.67	12.33	12.11
1653	185.26	223.25	206.67	18.38	16.98	16.75
1671	193.10	2.68	139.75	12.45	11.26	11.61
1677	191.90	-11.19	323.46	10.34	9.11	9.22

1702	158.72	183.18	169.45	6.98	6.42	6.05
1784	187.76	157.31	178.96	4.51	3.62	3.37
1786	157.99	213.74	169.31	16.31	13.10	13.69
1794	161.13	33.91	327.28	19.33	16.50	16.48
1814	168.78	-3.32	305.14	16.60	16.36	16.33
1823	146.61	12.20	18.11	18.00	17.63	16.75
1827	150.42	15.50	155.40	18.28	16.72	16.60
1860	176.94	164.52	-8.65	2.35	2.31	1.81
1874	190.04	186.40	63.33	20.02	20.01	19.77
1881	200.94	12.86	175.09	7.21	6.56	5.92
1902	188.09	36.61	163.31	11.73	9.13	9.66
1909	181.16	173.22	197.46	2.15	1.75	1.69
1927	177.76	197.27	124.82	18.20	18.21	17.64
1944	161.63	225.73	166.20	21.31	19.19	19.92
1992	171.79	221.88	30.35	18.91	16.76	16.99
1996	163.68	140.98	183.59	15.10	13.51	13.36

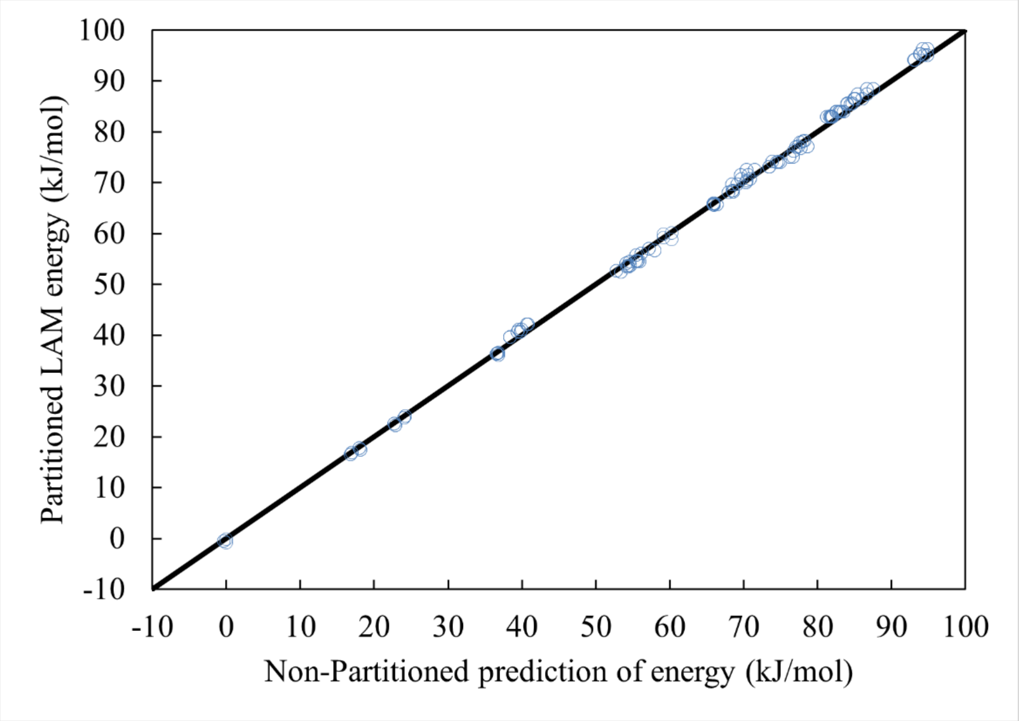

Figure 2. Parity plot showing the predicted values of 〖ΔU〗^intrawith the partitioned scheme, in kJ/mol, against the QM-calculated of 〖ΔU〗^intra at all 217 reference points in the non-partitioned scheme, in kJ/mol. The black line is the line for y=x

Section 3.2

Local approximate models (LAMs) were constructed by using a uniform grid along the DOFs, at 120° increments, before running the adaptive LAM algorithm[6] until converged to 5 kJ/mol accuracy

Molecule XX – Section 3.3

Local approximate models (LAMs) were constructed by using a uniform grid along the DOFs, at 120° increments, before running the adaptive LAM algorithm until converged to 5 kJ/mol accuracy, at the PBEPBE/6311G(d,p) level of theory. Following analysis and clustering, CrystalOptimizer[5] was used to refine the structures within 20 kJ/mol from the global minimum, at the PBE0/6-31G(d,p) level of theory, with extra flexibility introduced (angles around previously flexible torsions).

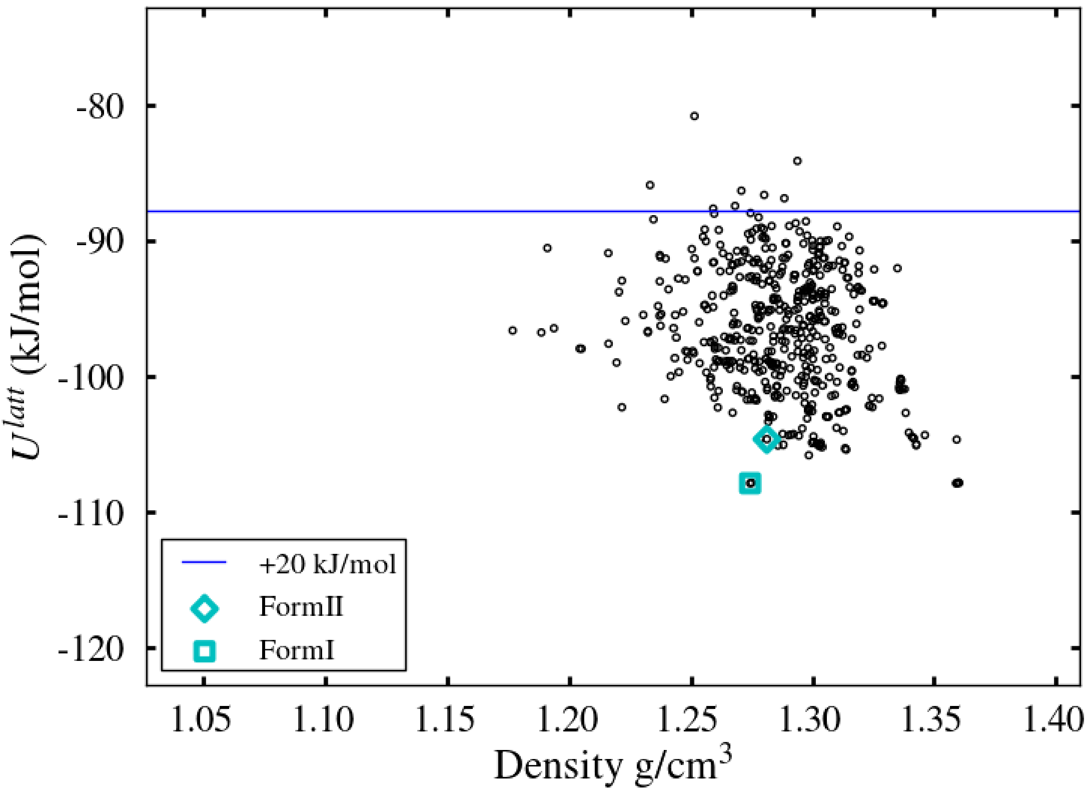

Figure 3. Polymorphic landscape for paracetamol after refinement stage with CrystalOptimizer

Safinamide – Section 3.4

Local approximate models (LAMs) were constructed by using a uniform grid along the DOFs, at 120° increments, before running the adaptive LAM algorithm[3] until converged to 5 kJ/mol accuracy, at the PBE/6311G(d,p) level of theory. Following analysis and clustering, CrystalOptimizer[5] was used to refine the structures within 20 kJ/mol from the global minimum, at the PBE0/6-311G(d,p) level of theory, with extra flexibility introduced (angles around previously flexible torsions).

During the multipole refinement stage, CSO-FM[7], a new multipole based local minimisation program, was used in place of CrystalOptimizer. It was found that by using a generic, transferable force-field like FIT, it was not possible to achieve a satisfactory geometry match and energy re-ranking. Instead, a custom set of force-field parameters was fitted using the parameter fitting methodology described in Bowskill (2021). In summary, periodic-DFT-D calculations were used to produce high-quality reference data (lattice energies and geometries) for 445 training structures, which included both hydrogen bonding and non-hydrogen bonding structures. The training structures consisted of small molecules and did not include safinamide. Repulsion-dispersion parameters were then regressed to this reference data, with the objective to minimise the relative error in geometry and energy at lattice-energy minima. Crucially, during the parameter estimation, all lattice-energy minimisation

calculations relied on multipoles derived at the same level of theory and basis set as used in the safinamide refinement (PBE0/6-311G(d,p)). This ensures that the Buckingham potential parameters used in the safinamide refinement are exactly tailored to the QM theory utilised. The optimised parameters are given in the following table:

Table 2. Parameters used in safinamide refinement. Atom symbols are element name then 2 underscores, except for Hydrogen bonded to a non polar atom (H_c) and Hydrogen bonded to a polar atom (H_p)

atom 1	atom 2	A (eV)	B (Å^{-1})	C (eV/Å^6)
C__	C__	3408.136	0.277778	24.84392
C__	H_c	477.0672	0.27248	5.007672
C__	O__	2330.978	0.26455	13.78847
C__	N__	2717.349	0.271003	18.86191
C__	F__	2626.9	0.257732	9.658888
C__	Cl_	6547.483	0.279525	45.56436
C__	S__	4189.577	0.289855	51.50225
C__	H_p	361.5044	0.242131	0
H_c	H_c	66.77934	0.26738	1.009373
H_c	O__	326.2878	0.25974	2.779277
H_c	N__	380.3715	0.265957	3.801906
H_c	F__	367.7106	0.253165	1.946897
H_c	Cl_	916.5095	0.27416	9.184196
H_c	S__	586.4523	0.284091	10.38107
H_c	H_p	50.60299	0.238095	0
O__	O__	1594.261	0.252525	7.652652
O__	N__	1858.518	0.258398	10.46843
O__	F__	1796.656	0.246305	5.36072
O__	Cl_	4478.12	0.266134	25.28839
O__	S__	2865.441	0.275482	28.58394
O__	H_p	247.2492	0.232019	0
N__	N__	2166.575	0.26455	14.32027
N__	F__	2094.46	0.251889	7.333185
N__	Cl_	5220.388	0.272665	34.59321
N__	S__	3340.4	0.282486	39.10134
N__	H_p	288.2319	0.236967	0
F__	F__	2024.745	0.240385	3.75521
F__	Cl_	5046.625	0.259235	17.71464
F__	S__	3229.214	0.268097	20.02319

F__	H_p	278.6379	0.226757	0
Cl_	Cl_	12578.59	0.281294	83.56618
Cl_	H_p	694.4981	0.243457	0
S__	S__	5150.191	0.30303	106.7658
S__	H_p	444.3926	0.251256	0
H_p	H_p	38.34513	0.214592	0

Computational Resources

The majority of the calculations reported here used the Imperial College HPC service, on the following hardware: https://icl-rcs-user-guide.readthedocs.io/en/latest/hpc/cluster-specification/

(1) Frisch, M. J.; Trucks, G. W.; Schlegel, H. B.; Scuseria, G. E.; Robb, M. A.; Cheeseman, J. R.; Scalmani, G.; Barone, V.; Petersson, G. A.; Nakatsuji, H.; Li, X.; Caricato, M.; Marenich, A. V.; Bloino, J.; Janesko, B. G.; Gomperts, R.; Mennucci, B.; Hratchian, H. P.; Ortiz, J. V.; Izmaylov, A. F.; Sonnenberg, J. L.; Williams; Ding, F.; Lipparini, F.; Egidi, F.; Goings, J.; Peng, B.; Petrone, A.; Henderson, T.; Ranasinghe, D.; Zakrzewski, V. G.; Gao, J.; Rega, N.; Zheng, G.; Liang, W.; Hada, M.; Ehara, M.; Toyota, K.; Fukuda, R.; Hasegawa, J.; Ishida, M.; Nakajima, T.; Honda, Y.; Kitao, O.; Nakai, H.; Vreven, T.; Throssell, K.; Montgomery Jr., J. A.; Peralta, J. E.; Ogliaro, F.; Bearpark, M. J.; Heyd, J. J.; Brothers, E. N.; Kudin, K. N.; Staroverov, V. N.; Keith, T. A.; Kobayashi, R.; Normand, J.; Raghavachari, K.; Rendell, A. P.; Burant, J. C.; Iyengar, S. S.; Tomasi, J.; Cossi, M.; Millam, J. M.; Klene, M.; Adamo, C.; Cammi, R.; Ochterski, J. W.; Martin, R. L.; Morokuma, K.; Farkas, O.; Foresman, J. B.; Fox, D. J. Gaussian 16 Rev. C.01, 2016.

(2) Habgood, M.; Sugden, I. J.; Kazantsev, A. V.; Adjiman, C. S.; Pantelides, C. C. Efficient Handling of Molecular Flexibility in Ab Initio Generation of Crystal Structures. *Journal of chemical theory and computation* **2015**, *11* (4), 1957–1969.

(3) Sugden, I. J.; Adjiman, C. S.; Pantelides, C. C. Accurate and Efficient Representation of Intramolecular Energy in Ab Initio Generation of Crystal Structures. II. Smoothed Intramolecular Potentials. *Acta Crystallographica Section B: Structural Science, Crystal Engineering and Materials* **2019**, *75* (3), 423–433.

(4) Williams, D. t; Cox, S. R. Nonbonded Potentials for Azahydrocarbons: The Importance of the Coulombic Interaction. *Acta Crystallographica Section B: Structural Science* **1984**, *40* (4), 404–417.

(5) Kazantsev, A. V.; Karamertzanis, P. G.; Adjiman, C. S.; Pantelides, C. C. Efficient Handling of Molecular Flexibility in Lattice Energy Minimization of Organic Crystals. *Journal of Chemical Theory and Computation* **2011**, *7* (6), 1998–2016.

(6) Sugden, I.; Adjiman, C. S.; Pantelides, C. C. Accurate and Efficient Representation of Intramolecular Energy in Ab Initio Generation of Crystal Structures. I. Adaptive Local Approximate Models. *Acta Crystallographica Section B: Structural Science, Crystal Engineering and Materials* **2016**, *72* (6), 864–874.

(7) David H. Bowskill. Reliable and Efficient Parameter Estimation Methodologies for Crystal Structure Prediction, Imperial College London, 2021.
